# Melatonin as a Signaling Molecule for Metabolism Regulation in Response to Hypoxia in the Crab *Neohelice granulata*

**DOI:** 10.3390/ijms151222405

**Published:** 2014-12-04

**Authors:** Fábio Everton Maciel, Márcio Alberto Geihs, Bruno Pinto Cruz, Marcelo Alves Vargas, Silvana Allodi, Luis Fernando Marins, Luiz Eduardo Maia Nery

**Affiliations:** 1 Programa de Pós-Graduação em Ciências Fisiológicas—Fisiologia Animal Comparada, Instituto de Ciências Biológicas, Universidade Federal do Rio Grande (FURG), 96201-300 Rio Grande, Brazil; E-Mails: geihs@hotmail.com (M.A.G.); brunopintocruz@hotmail.com (B.P.C.); biovargas@gmail.com (M.A.V.); dqmluf@furg.br (L.F.M.); famnery@terra.com.br (L.E.M.N.); 2 Programa de Pós-Graduação em Ciências Morfológicas, Instituto de Ciências Biomédicas, Centro de Ciências da Saúde, Universidade Federal do Rio de Janeiro, 21949-902 Rio de Janeiro, Brazil; E-Mail: sallodi@biof.ufrj.br

**Keywords:** melatonin, Decapoda, hypoxia, metabolism, glucose, lactate, crustacean hyperglycemic hormone (CHH)

## Abstract

Melatonin has been identified in a variety of crustacean species, but its function is not as well understood as in vertebrates. The present study investigates whether melatonin has an effect on crustacean hyperglycemic hormone (CHH) gene expression, oxygen consumption (VO_2_) and circulating glucose and lactate levels, in response to different dissolved-oxygen concentrations, in the crab *Neohelice granulata*, as well as whether these possible effects are eyestalk- or receptor-dependent. Melatonin decreased CHH expression in crabs exposed for 45 min to 6 (2, 200 or 20,000 pmol·crab^−1^) or 2 mgO_2_·L^−1^ (200 pmol·crab^−1^). Since luzindole (200 nmol·crab^−1^) did not significantly (*p* > 0.05) alter the melatonin effect, its action does not seem to be mediated by vertebrate-typical MT1 and MT2 receptors. Melatonin (200 pmol·crab^−1^) increased the levels of glucose and lactate in crabs exposed to 6 mgO_2_·L^−1^, and luzindole (200 nmol·crab^−1^) decreased this effect, indicating that melatonin receptors are involved in hyperglycemia and lactemia. Melatonin showed no effect on VO_2_. Interestingly, *in vitro* incubation of eyestalk ganglia for 45 min at 0.7 mgO_2_·L^−1^ significantly (*p* < 0.05) increased melatonin production in this organ. In addition, injections of melatonin significantly increased the levels of circulating melatonin in crabs exposed for 45 min to 6 (200 or 20,000 pmol·crab^−1^), 2 (200 and 20,000 pmol·crab^−1^) and 0.7 (200 or 20,000 pmol·crab^−1^) mgO_2_·L^−1^. Therefore, melatonin seems to have an effect on the metabolism of *N. granulata*. This molecule inhibited the gene expression of CHH and caused an eyestalk- and receptor-dependent hyperglycemia, which suggests that melatonin may have a signaling role in metabolic regulation in this crab.

## 1. Introduction

Half a century ago, Lerner *et al.* [1,2] isolated from thousands of bovine pineal glands, *N*-acetyl-5-methoxytryptamine or melatonin. Its name comes from the Greek *melano* = black and *tonos* = color, due to its lightening effect on frog skin. The main action of this molecule, at least in vertebrates, is as a signal of the photoperiod length, which allows organisms to better adapt to and also to anticipate periodic environmental changes [3,4,5]. This indoleamine is present in a variety of organisms, from unicellular algae to mammals, and its production follows a circadian rhythm [6,7]. Melatonin has also been identified in crustaceans, although its production and functions are not as well understood as they are in vertebrates [8,9]. Maciel and colleagues [10] reported that when the gills of the crab *Neohelice granulata* were incubated with melatonin (20 × 10^−9^ M), oxygen consumption decreased. Tilden *et al.* [11] observed a shift in the circadian rhythm of glucose and lactate in the hemolymph of melatonin-injected *Uca pugilator* (3 × 10^−9^ mol·animal^−1^), with one peak in the middle of the light phase. In another study, Sainath and Reddy [12] demonstrated a dose-dependent effect of melatonin on hyperglycemia in the crab *Oziotelphusa senex senex*. These reports suggest that this indoleamine may have a role in metabolic regulation in crustaceans.

Since glucose is one of the main energy substrates for the general metabolic processes in crustaceans [13], several studies have analyzed the circulating glucose levels in relation to various environmental stresses imposed on the animals, such as temperature [14,15,16,17,18], salinity [19,20] and hypoxia [21,22,23,24,25]. The general response to these stressors is hyperglycemia, evoked by the crustacean hyperglycemic hormone (CHH). This hormone is a peptide that has been identified mainly in the neuroendocrine system localized in the optic ganglia of eyestalk (x-organ/sinus gland complex), but also in other sites such as cerebral ganglia, ventral nerve cord, gill, hindgut and others (for review see [26]). The CHH nucleotide gene sequence was determined in a variety of crustacean species, and its main action is the modulation of hemolymth glucose levels through carbohydrate mobilization [26]. However, the control of synthesis and release of this peptide are not completely understood.

The estuarine crab *Neohelice granulata* has a high tolerance to hypoxia and anoxia, conditions related to decreased dissolved-oxygen content in the water or to air exposure [27,28], common situations in salt marshes, their natural habitat [29]. Specific metabolic adjustments, such as decreasing locomotor activity and oxygen consumption (VO_2_), and increasing glucose and lactate levels in the hemolymph, allow this crab to tolerate these stressors [21,28,30,31,32]. Maciel *et al.* [25] found that severe hypoxia (45 min at 0.7 mgO_2_·L^−1^) induced a sharp decrease of VO_2_ and an increase of circulating lactate in both intact and eyestalkless crabs, but hyperglycemia only in intact ones, indicating a dependence on eyestalk CHH for this response. As some of the above-mentioned studies indicate that melatonin is involved in the metabolic regulation of crustaceans, the present study evaluated whether melatonin is capable of inducing metabolic changes similar to those that occur during hypoxia, and if these responses are eyestalk- and melatonin receptor-dependent.

## 2. Results

**Figure 1:**
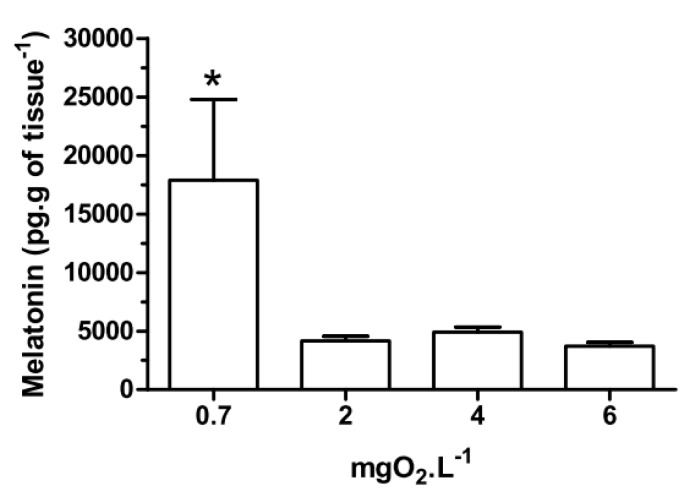
Melatonin content in the eyestalks of the crab *Neohelice granulata in vitro*. The eyestalks were incubated for 45 min at 0.7, 2, 4 or 6 mgO_2_·L^−1^. Each point represents the mean ± standard error (SE) (*n* = 5). * represents a significant difference (*p* < 0.05) from the other groups.

**Figure 2:**
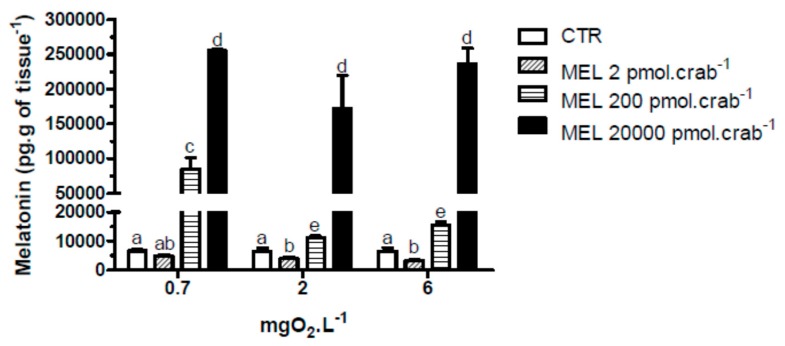
Melatonin content in the eyestalks of the crab *Neohelice granulata* exposed for 45 min to three different dissolved-oxygen concentrations (0.7, 2 or 6 mgO_2_·L^−1^) and injected with physiological saline (control group—CTR) or melatonin (2, 200 or 20,000 pmol·crab^−1^). Each point represents the mean ± SE (*n* = 3–4). Different letters represent significant differences (*p* < 0.05) between the groups.

With respect to melatonin levels, eyestalk ganglia incubated for 45 min at 0.7 mgO_2_·L^−1^ (Figure 1), showed a significant (*p* < 0.05) increase (17,886 ± 6908 pg·g of tissue^−1^) compared to 2, 4 and 6 mgO_2_·L^−1^ (4165 ± 397, 4907 ± 428 and 3709 ± 334 pg·g of tissue^−1^, respectively). Interestingly, in the *in vivo* experiment, crabs treated with 20,000 pmol·crab^−1^ had a higher eyestalk melatonin content (*p* < 0.05) in all three levels of oxygen incubation (255,206 ± 2129 pg·g of tissue^−1^ for 0.7 mgO_2_·L^−1^; 171,409 ± 37,423 pg·g of tissue^−1^ for 2 mgO_2_·L^−1^; and 235,900 ± 20,693 pg·g of tissue^−1^ for 6 mgO_2_·L^−1^) compared to the other treatment groups. Crabs treated with 200 pmol·crab^−1^ and exposed for 45 min at 0.7 mgO_2_·L^−1^ showed a higher melatonin content in the eyestalks (84,831 ± 16,511 pg·g of tissue^−1^) compared with crabs exposed to 2 (11,273 ± 489 pg·g of tissue^−1^) and 6 (15,429 ± 1258 pg·g of tissue^−1^) mgO_2_·L^−1^ under the same treatment, and also compared to crabs treated with 2 pmol·crab^−1^ and physiological saline (see Figure 2).

**Figure 3:**
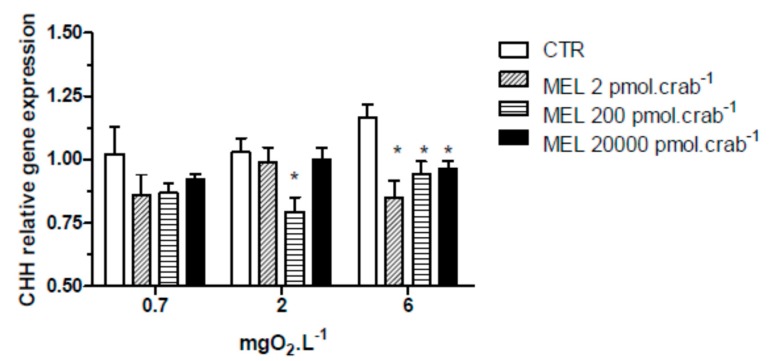
Crustacean Hyperglycemic Hormone (CHH) relative gene expression in the eyestalks of *Neohelice granulata* exposed for 45 min to three different dissolved-oxygen concentrations (0.7, 2 or 6 mgO_2_·L^−1^) and injected with physiological saline (CTR) or melatonin (2, 200 or 20,000 pmol·crab^−1^). Each point represents the mean ± SE (*n* = 3–4). * represents a significant difference (*p* < 0.05) from the CTR group. The values are normalized for β-actin gene expression.

All three doses of melatonin tested were capable of significantly (*p* < 0.05) decreasing CHH gene expression (Figure 3) in the eyestalks of crabs exposed to 6 mgO_2_·L^−1^ (0.85 ± 0.07, 0.94 ± 0.04 and 0.96 ± 0.03 relative expression for 2, 200 and 20,000 pmol·crab^−1^ of melatonin, respectively), compared to the control group (1.16 ± 0.06 relative expression). In crabs exposed to 2 mgO_2_·L^−1^, the dose of 200 pmol·crab^−1^ of melatonin significantly (*p* < 0.05) decreased (0.79 ± 0.03 relative expression) the CHH gene expression compared to the control group (1.03 ± 0.05 relative expression). No significant differences (*p* > 0.05) were observed between the melatonin doses and the control group in crabs exposed to 0.7 mgO_2_·L^−1^.

**Figure 4:**
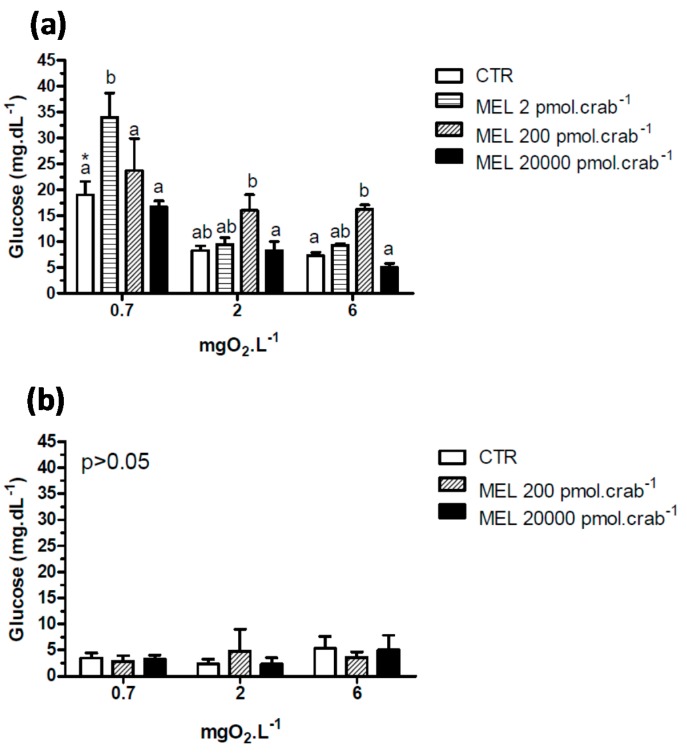
Circulating glucose concentration in (**a**) intact and (**b**) eyestalkless *Neohelice granulata* exposed for 45 min to three different dissolved-oxygen concentrations (0.7, 2 or 6 mgO_2_·L^−1^) and injected with physiological saline (CTR) or melatonin (2, 200 or 20,000 pmol·crab^−1^). Each point represents the mean ± SE (*n* = 4–7). Different letters represent significant differences (*p* < 0.05) between the groups with the same dissolved oxygen concentration. Different letters represent significant differences (*p* < 0.05) between the groups.

A significant hyperglycemia (*p* < 0.05) was observed in intact crabs (Figure 4a) exposed to 6 mgO_2_·L^−1^ and injected with 200 pmol·crab^−1^ of melatonin (16.2 ± 0.87 mg·dL^−1^) compared to the control group (7.3 ± 0.7 mg·dL^−1^). This effect was not significant (*p* > 0.05) in crabs exposed to 2 mgO_2_·L^−1^. In crabs exposed to 0.7 mgO_2_·L^−1^, a significant (*p* < 0.05) hyperglycemia was observed in the control group (19 ± 2.6 mg·dL^−1^) compared to the same group exposed to 6 (7.3 ± 0.7 mg·dL^−1^) and 2 (8.2 ± 0.9 mg·dL^−1^) mgO_2_·L^−1^. Interestingly, glucose levels increased significantly (*p* < 0.05) in crabs exposed to 0.7 and injected with 2 pmol·crab^−1^ of melatonin (33.9 ± 4.7 mg·dL^−1^). Eyestalkless crabs (Figure 4b) showed no significant differences (*p* > 0.05) in all dissolved-oxygen concentrations and melatonin doses injected.

**Figure 5:**
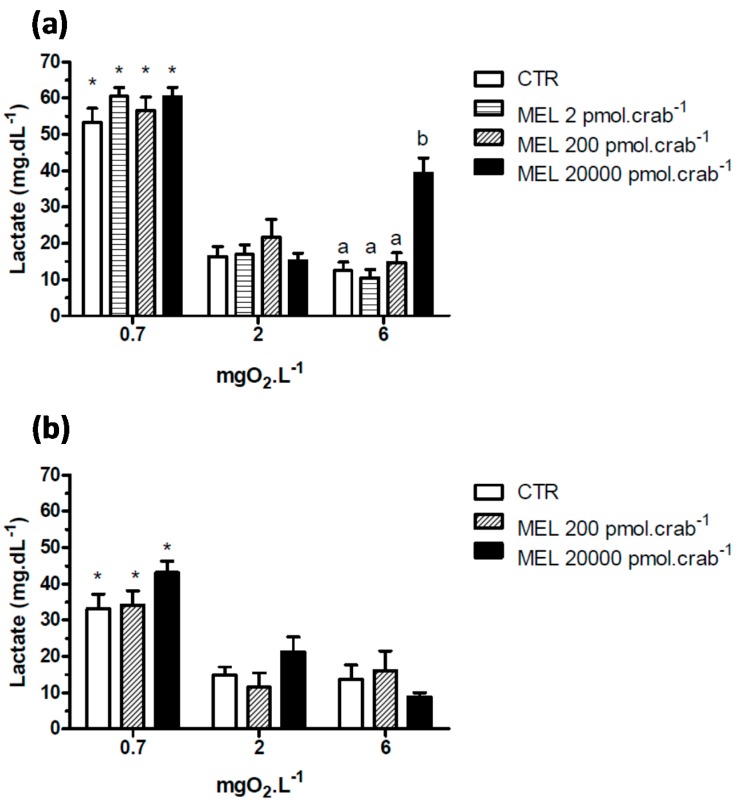
Circulating lactate in (**a**) intact and (**b**) eyestalkless *Neohelice granulata* exposed for 45 min to three different dissolved oxygen concentrations (0.7, 2 or 6 mgO_2_·L^−1^) and injected with physiological saline (CTR) or melatonin (2, 200 or 20,000 pmol·crab^−1^). Each point represents the mean ± SE (*n* = 4–7). Different letters represent significant differences (*p* < 0.05) between the groups with the same dissolved oxygen concentration. * represents a significant difference (*p* < 0.05) from the CTR group at 6 mgO_2_·L^−1^.

Intact crabs exposed to 6 mgO_2_·L^−1^ and injected with 20,000 pmol·crab^−1^ of melatonin showed a significant (*p* < 0.05) increase in lactate (Figure 5a) concentration (39.3 ± 4.2 mg·dL^−1^) compared to the control group (12.5 ± 2.1 mg·dL^−1^). No significant effects (*p* > 0.05) were observed in crabs exposed to 2 mgO_2_·L^−1^ and injected with the doses of melatonin. However, when the crabs were exposed to 0.7 mgO_2_·L^−1^, all groups showed a significant increase (*p* < 0.05) in lactate concentration compared to the other dissolved-oxygen concentrations. In eyestalkless crabs (Figure 5b) only the exposure to 0.7 mgO_2_·L^−1^ significantly increased (*p* < 0.05) lactate levels in all treatment groups.

**Figure 6:**
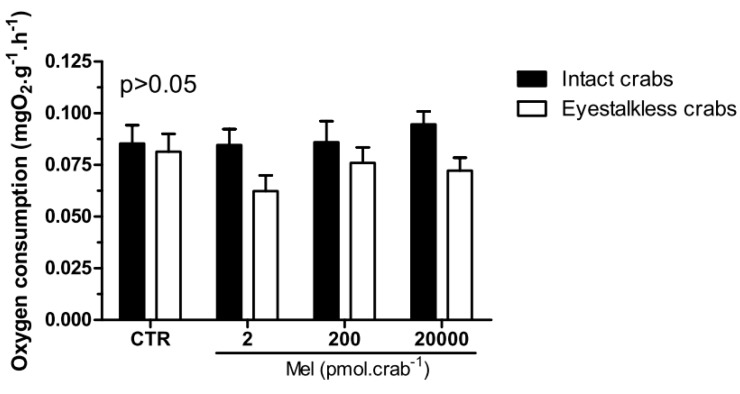
Oxygen consumption (VO_2_) of intact (black bars) and eyestalkless (open bars) *Neohelice granulata* exposed for 45 min only to 6 mgO_2_·L^−1^ and injected with physiological saline (CTR) or melatonin (2, 200 or 20,000 pmol·crab^−1^). Each point represents the mean ± SE (*n* = 3–6). No significant differences (*p* > 0.05) were observed.

Intact and eyestalkless (Figure 6) crabs exposed to 6 mgO_2_·L^−1^ and treated with melatonin showed no significant differences (*p* > 0.05) in VO_2_.

**Figure 7:**
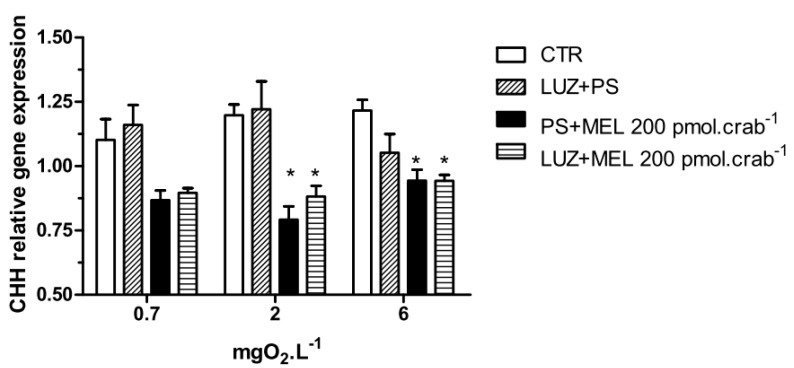
Crustacean Hyperglycemic Hormone (CHH) relative gene expression in the eyestalks of *Neohelice granulata* exposed for 45 min to three different dissolved oxygen concentrations (0.7, 2 or 6 mgO_2_·L^−1^) and injected with physiological saline only (CTR), luzindole (200 nmol·crab^−1^) plus physiological saline, physiological saline plus melatonin (200 pmol·crab^−1^), or luzindole (200 nmol·crab^−1^) plus melatonin (200 pmol·crab^−1^). Each point represents the mean ± SE (*n* = 3–4). * represents a significant difference (*p* < 0.05) from the CTR. The values are normalized for β-actin gene expression.

Melatonin (200 pmol·crab^−1^) significantly decreased (*p* < 0.05) the CHH gene expression in crabs exposed to 6 (0.94 ± 0.02 relative expression) and 2 (0.8 ± 0.05 relative expression) mgO_2_·L^−1^ independently of whether they were injected with luzindole (200 nmol·crab^−1^), compared to the control group (1.21 ± 0.04 and 1.19 ± 0.04 relative expression, respectively) (Figure 7). No significant differences were observed in animals exposed to 0.7 mgO_2_·L^−1^.

**Figure 8:**
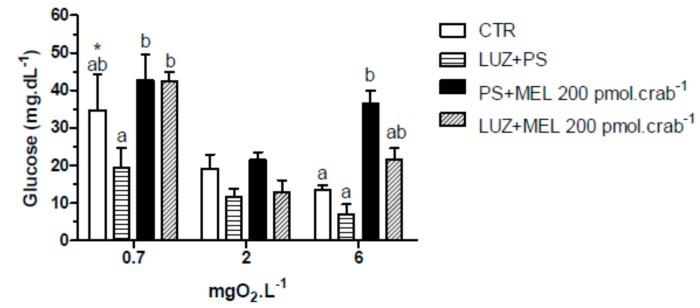
Circulating glucose concentration in intact *Neohelice granulata* exposed for 45 min to three different dissolved-oxygen concentrations (0.7, 2 or 6 mgO_2_·L^−1^) and injected with physiological saline only (CTR), luzindole (200 nmol·crab^−1^) plus physiological saline, physiological saline plus melatonin (200 pmol·crab^−1^), or luzindole (200 nmol·crab^−1^) plus melatonin (200 pmol·crab^−1^). Each point represents the mean ± SE (*n* = 4–6). Different letters represent significant differences (*p* < 0.05) between the groups with the same dissolved oxygen concentration. * represents a significant difference (*p* < 0.05) from the CTR group at 6 mgO_2_·L^−1^.

Again, injections of melatonin (200 pmol·crab^−1^) significantly increased (*p* < 0.05) glucose levels in intact crabs (Figure 8) exposed to 6 mgO_2_·L^−1^ (36.4 ± 3.5 mg·dL^−1^); but crabs that were also injected with luzindole (200 nmol·crab^−1^) did not differ (*p* > 0.05) from the control group (21.5 ± 3.5 and 3.4 ± 1.3 mg·dL^−1^, respectively). No significant effects (*p* > 0.05) of melatonin or luzindole were observed in crabs exposed to 2 mgO_2_·L^−1^. In 0.7 mgO_2_·L^−1^ a general increase of glucose levels was observed in the treatment groups (34.7 ± 9.5 mg·dL^−1^, CTR as reference; *p* < 0.05 compared to CTR of 6 mgO_2_·L^−1^).

**Figure 9:**
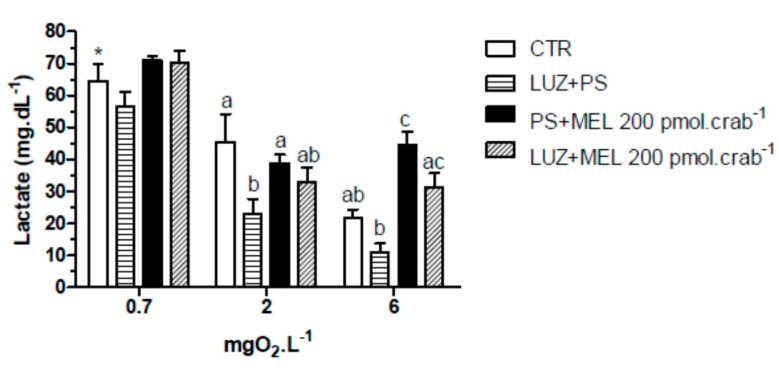
Circulating lactate concentration in intact *Neohelice granulata* exposed for 45 min to three different dissolved-oxygen concentrations (0.7, 2 or 6 mgO_2_·L^−1^) and injected with physiological saline only (CTR), luzindole (200 nmol·crab^−1^) plus physiological saline, physiological saline plus melatonin (200 pmol·crab^−1^), or luzindole (200 nmol·crab^−1^) plus melatonin (200 pmol·crab^−1^). Each point represents the mean ± SE (*n* = 4–6). Different letters represent significant differences (*p* < 0.05) between the groups with the same dissolved oxygen concentration. * represents a significant difference (*p* < 0.05) from the CTR group at 6 mgO_2_·L^−1^.

Similar effects were observed in the lactate measurements (Figure 9). Melatonin (200 pmol·crab^−1^) significantly increased (*p* < 0.05) lactate concentration in crabs exposed to 6 mgO_2_·L^−1^ (44.5 ± 4.2 mg·dL^−1^), but crabs also injected with luzindole (200 nmol·crab^−1^) (31.5 ± 4.8 mg·dL^−1^) did not differ (*p* > 0.05) from the control group (21.8 ± 2.9 mg·dL^−1^). At 2 mgO_2_·L^−1^ luzindole significantly decreased (*p* < 0.05) lactate levels (23.1 ± 5.2 mg·dL^−1^) compared to the control group (45.5 ± 6.1 mg·dL^−1^) but did not significantly affect the melatonin group. Crabs exposed to 0.7 mgO_2_·L^−1^ showed a general lactemia in all treatment groups (64.5 ± 6.1 mg·dL^−1^, CTR as reference; *p* < 0.05 compared to CTR of 6 mgO_2_·L^−1^).

**Figure 10:**
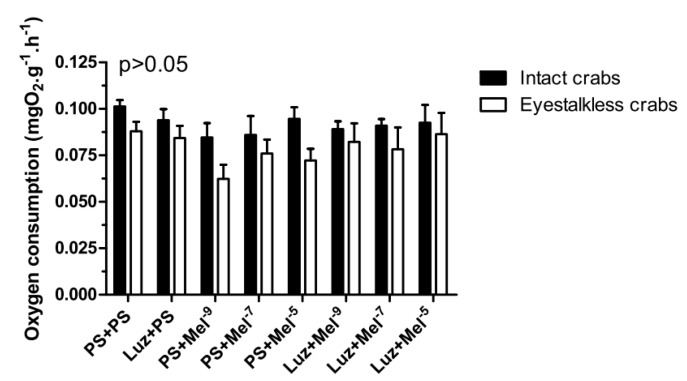
Oxygen consumption (VO_2_) of intact (black bars) and eyestalkless (open bars) *Neohelice granulata* exposed for 45 min only to 6 mgO_2_·L^−1^ and injected with physiological saline only (CTR) luzindole (200 nmol·crab^−1^) plus physiological saline, physiological saline plus melatonin (200 pmol·crab^−1^), or luzindole (200 nmol·crab^−1^) plus melatonin (200 pmol·crab^−1^). Each point represents the mean ± SE (*n* = 3–4). No significant differences (*p* > 0.05) were observed.

In the VO_2_ measurements, no significant differences (*p* > 0.05) were observed between the treatment groups in intact and eyestalkless (Figure 10) crabs exposed to 6 mgO_2_·L^−1^.

## 3. Discussion

As mentioned above, melatonin is ubiquitous in living organisms. In crustaceans, melatonin has been identified in a dozen species [8,9], but its function is unclear. Recent studies have shown an association of this molecule with metabolism in crabs. Maciel and co-workers [10] found that gills of *N. granulata* decreased their VO_2_ when incubated for 120 min with 20 × 10^−9^ M of melatonin, and also decreased gill VO_2_ in eyestalkless crabs injected with melatonin after 390 min. In contrast, when an equivalent dose of melatonin was injected in the same species, no effect was observed in muscle VO_2_ [33]. In the present study, melatonin did not cause any change in overall VO_2_ in both intact and eyestalkless crabs. Taken together, these data suggest that this indoleamine effect may be tissue-specific for depression of aerobic metabolism, but as muscle tissue is the most important, the overall VO_2_ in this crab is not significantly affected by melatonin, at least in the dosages tested.

In addition to aerobic metabolism, melatonin seems to have a role in glucose/lactate effects in crustaceans. In eyestalkless *Uca pugilator*, injections of melatonin (3 × 10^−9^ mol·animal^−1^) caused an increase in glucose and lactate levels, indicating an independence of this effect of CHH from the eyestalk [11]. In this case, melatonin could act as a secretagogue of other CHH-releasing tissue and/or act directly in the breakdown of glycogen to glucose through glycogen phosphorylase in the tissues. Sainath and Reddy [12] observed an increase of phosphorylase activity in hepatopancreas and muscle of intact and eyestalkless crab *Oziotelphusa senex senex* when injected with melatonin (10^−7^ mol·crab^−1^), and also hyperglycemia in a dose-dependent manner (10^−10^–10^−5^ mol·crab^−1^), which supports the hypothesis that melatonin-induced hyperglycemia is not through CHH from the eyestalk, at least in this species. Here, however, a hyperglycemia was observed in intact but not in eyestalkless *N. granulata* injected with 200 pmol·crab^−1^, indicating a dependence on CHH from the eyestalks. In addition, this effect seems to occur through melatonin receptors, since with the administration of luzindole (200 nmol·crab^−1^), glucose levels did not significantly differ from the control group.

The effect of melatonin on hyperglycemia in *N. granulata* is affected by hypoxia. In severe hypoxia (0.7 mg·L^−1^), the lowest melatonin dose (2 pmol·crab^−1^) caused hyperglycemia, but 200 pmol·crab^−1^ did not. The hypoxic condition seems to increase sensitization to melatonin effects, suggesting that melatonin in low doses could mobilize carbohydrates.

Melatonin (20,000 pmol·crab^−1^) also increased lactate levels in *N. granulata*, but only when the crabs were exposed to 6 mgO_2_·L^−1^. In severe hypoxia, the lactate concentration increased significantly, independently of whether the crabs were treated with melatonin or not. In this species, some studies have found that lactate is the main metabolite of anaerobic metabolism. In addition, the lactate level increases when the crab is exposed to hypoxic or anoxic situations [23,25,32]. However, this effect is not totally eyestalk-dependent, since eyestalkless crabs showed a lower but still significant increase in lactate levels.

Interestingly, melatonin decreased CHH gene expression when *N. granulata* was exposed for 45 min to 6 (2, 200 and 20,000 pmol·crab^−1^) and 2 (200 pmol·crab^−1^) mgO_2_·L^−1^, but its effect seems to occur through another pathway than the melatonin receptor. This downregulation of the CHH gene expression by melatonin could be an indirect effect of this indoleamine and a negative feedback response to the increase in circulating glucose levels. Chung and Zmora [34] also observed an inhibition of CHH expression in the blue crab *Callinectes sapidus*, but in response to 1 h of hypoxia conditions. Again, a negative feedback could be regulating this gene in response to the hyperglycemia seen in *C. sapidus* under hypoxic conditions [34]. However, this effect was not observed in *N. granulata* [25], perhaps because this species is more tolerant to hypoxia or anoxia than *C. sapidus*, and 45 min was not sufficient to induce downregulation in eyestalk CHH expression.

In addition to the various functions previously described for melatonin, this molecule has the ability, at least in mammals, to influence insulin secretion, and metabolism of lipids and glucose [35,36,37,38,39]. Additionally, some studies have observed that this indoleamine may modulate the release of neurohormones in insects, such as the prothoracicotropic hormone in *Periplaneta americana* [40] and the adipokinetic precursor-related peptide in *Locusta migratoria* [41]. The only similar study on crustaceans was conducted by Tilden* et al.* [42], who observed an increase in synaptic transmission at the neuromuscular junction when melatonin was injected into *Procambarus clarkii*. In the present study, melatonin induced hyperglycemia, increased circulating lactate, and inhibited CHH gene expression. This indoleamine could be stimulating CHH release, and as a consequence, the higher levels of glucose could inhibit CHH gene expression; or perhaps the CHH peptide regulates its transcription.

Melatonin membrane receptors have been identified in vertebrates, and are classified as MT1, MT2 and MT3 [43,44]. MT1 and MT2 are G protein-coupled receptors, and MT3 is an enzyme belonging to the family of quinone reductases, although its signaling pathway is still unknown. In invertebrates, melatonin receptors are little understood. Richter *et al.* [40] found that luzindole (10 nM) inhibited the prothoracicotropic hormone from the brain of *P. americana* when incubated with melatonin (10 nM). Concerning crustaceans, Mendoza-Vargas [45] observed an inhibition of the receptor potential amplitude of retinular photoreceptors by DH97 (1 nM), a MT_2_ receptor-selective antagonist, whereas melatonin (100 nM) increased the receptor potential amplitude in the crayfish *P. clarkii*. Here, luzindole (200 nmol·crab^−1^) inhibited the increase in glucose and lactate caused by melatonin (200 pmol·crab^−1^), but did not affect the CHH gene expression in *N. granulata*. Thus, melatonin may have different modes of action in the regulation of carbohydrate metabolism, since the melatonin response was seen at transcriptional and physiological levels.

Eyestalks incubated for 45 min at 2, 4 or 6 mgO_2_·L^−1^ showed similar levels of melatonin, as previously reported [8]. However, at 0.7 mgO_2_·L^−1^ the melatonin content increased (Figure 1). This response was seen only *in vitro*, not in *in vivo* experiments, suggesting that melatonin may be a signaling molecule in this tissue only under conditions of severe hypoxia. Interestingly, injection of melatonin increased the melatonin content in the eyestalks of crabs exposed to different dissolved-oxygen concentrations, in a dose-dependent manner (see Figure 2). But, in animals injected with 200 pmol·crab^−1^ and exposed to 0.7 mgO_2_·L^−1^, the melatonin content in the eyestalks was higher than in the other groups. Again, these results reinforce the suggestion that melatonin might be involved in metabolic regulation in this crab when it undergoes hypoxic conditions.

In conclusion, melatonin seems to induce hyperglycemia and anaerobic metabolism in *N. granulata,* since glucose and lactate are augmented, but these responses are eyestalk- and melatonin receptor-dependent. Also, melatonin down regulates CHH gene expression, but not by a known melatonin receptor mechanism.

## 4. Material and Methods

### 4.1. Animals

Adult male crabs of *N. granulata* (mean weight ± confidence interval: 11.2 ± 1.1 g) were collected in salt marshes near the city of Rio Grande in southern Brazil. In the laboratory, the crabs were acclimated under constant conditions of salinity (20), temperature (20 °C) and photoperiod (12L:12D) for at least 7 days. During this period, the crabs were fed regularly with ground beef.

### 4.2. Reagents

Melatonin (MEL) and luzindole (LUZ) were purchased from Sigma-Aldrich (St. Louis, MO, USA). Stock solutions were prepared with ethanol and dimethyl sulfoxide (DMSO), respectively. Working solutions were prepared with crustacean physiological solution (PS), which contained: 1 × 10^−2^ M MgCl_2_, 355 × 10^−3^ M NaCl, 16.6 × 10^−3^ M CaCl_2_, 5 × 10^−3^ M H_3_BO_3_, 1 × 10^−2^ M KHCO_3_ and 8 × 10^−3^ M sodium citrate (Na_3_C_6_H_5_O_7_·2H_2_O); pH adjusted to 7.6.

### 4.3. RNA Isolation and cDNA Synthesis

The eyestalk ganglia were collected, dissected, and used for total RNA extraction. Total RNA was isolated by the Trizol Reagent Solution™ method, following the manufacturer’s instructions (Applied Biosystems, Grand Island, NY, USA). Genomic DNA was degraded with DNase I (Invitrogen) and total RNA was quantified by a Qubit™ fluorometer using the Quant-iT™ RNA assay kit (Invitrogen, Waltham, MA, USA), and calibration was carried out using a two-point standard curve. First-strand cDNA was synthesized from approximately 1 μg of total RNA and used as a template with the SuperScript III enzyme kit (Invitrogen) according to the manufacturer’s suggested protocol.

### 4.4. Experimental Design and Measurements of CHH Gene Expression, VO_2_, Glucose and Lactate for Different O_2_ Concentrations and Melatonin Dose Effects

Twelve aquaria (3 L each) were purged with nitrogen gas to reduce the level of dissolved oxygen to 6 mgO_2_·L^−1^ (normoxia), 2 mgO_2_·L^−1^ (low hypoxia) or 0.7 mgO_2_·L^−1^ (severe hypoxia) (4 aquaria each). After that, the crabs (total of 192 animals) were injected with 100 μL of PS (control) or MEL (20 × 10^−9^, 20 × 10^−7^ or 20 × 10^−5^ M) and exposed for 45 min in one of the three oxygen concentrations, prior to eyestalk collection for CHH gene expression (*n* = 4, pool of 4 crabs) and hemolymph sampling (*n* = 5). An additional experiment was carried out with eyestalkless crabs (total of 45 animals) under the same conditions as for hemolymph sampling; animals had their eyestalks ablated (the eyestalks were cut off with scissors and then cauterized) 24 h prior to the beginning of the experiment. The eyestalk dissection and total RNA and cDNA synthesis were performed as described above. Quantitative analysis of CHH mRNA expression was carried out for 10 min at 95 °C and 40 cycles of 15 s at 95 °C and 1 min at 60 °C by real-time RT-PCR (7300 Real-Time System, Applied Biosystems), using SYBR GREEN PCR Master Mix™ (Applied Biosystems), CHH-specific primers (sense 5'-CGCACACGTGTTCCAGTTTG-3', and anti-sense 5'-AGATGCCCTTGCAGGAACG-3'; GenBank accession number ADM26761), Ultra Pure Distilled Water (Invitrogen) and 1.0 μL cDNA (dilution 1:19). Β-actin expression (sense 5'-CCAGATCATGTTTGAGGTGTTCA-3' and anti-sense 5'-GGGACAGCACAGCCTGGAT-3' primers; GenBank accession number AY074923) was used as an internal control to normalize the data.

Glucose and lactate were measured using the GOD-PAD monoreagent kit (Kovalent, Rio de Janeiro, Brazil) and lactate kit (Kovalent), respectively. Glucose and lactate are expressed in mg per dL of hemolymph.

For VO_2_ measurement, 9 aquaria (3 L each) were purged with nitrogen gas to reduce the level of dissolved oxygen to 6, 2 or 0.7 mgO_2_·L^−1^ (3 aquaria each). Thirty-six intact (*n* = 4) and 36 eyestalkless (*n* = 4) crabs were injected with 100 μL of PS (control) or MEL (20 × 10^−9^ or 20 × 10^−7^ M) and exposed to the above oxygen concentrations in the respirometer chamber with saline water (salinity at 20) for 15 min prior to the beginning of the experiment. Oxygen concentration was measured at time zero and after 30 min using a portable oxymeter. Oxygen consumption was expressed in milligrams of oxygen per gram of body weight per hour. The eyestalks were ablated as described above.

### 4.5. Experimental Design and Measurements of CHH Gene Expression, VO_2_, Glucose and Lactate for Different O_2_ Concentrations and Melatonin and Luzindole Dose Effects

Twelve aquaria (3 L each) were purged with nitrogen gas to reduce the level of dissolved oxygen to 6, 2 or 0.7 mgO_2_·L^−1^ (4 aquaria each). Next, the crabs (total of 192 animals) received two injections of 100 μL with a 15-min interval between them. One group received 2 injections of PS (control), the second group was injected first with LUZ (10^−4^ M) and then with PS, the third group was injected with PS and then with MEL (20 × 10^−7^ M), and the fourth group was injected first with LUZ (10^−4^ M) and then with MEL (20 × 10^−7^ M). Next, the crabs were exposed for 45 min to each oxygen concentration prior to eyestalk collection for CHH gene expression (*n* = 4, pool of 4 crabs) and hemolymph sampling (*n* = 5). An additional experiment was carried out with eyestalkless crabs (total of 45 animals) under the same conditions as for hemolymph sampling. The eyestalk dissection and analyses of total RNA, cDNA synthesis and CHH gene expression were performed as described above, as were the glucose and lactate measurements.

For VO_2_ measurement, 12 aquaria (3 L each) were purged with nitrogen gas to reduce the level of dissolved oxygen to 6 mgO_2_·L^−1^. Thirty-six intact (*n* = 4) and 36 eyestalkless (*n* = 4) crabs received two injections of 100 μL with a 15-min interval between them. The treatments were the same as described above: PS plus PS, LUZ (10^−4^ M) plus PS, PS plus MEL (20 × 10^−7^ M), or LUZ (10^−4^ M) plus MEL (20 × 10^−7^ M). After that, the animals were exposed to the above oxygen concentration in the respirometer chamber with saline water (salinity at 20) for 15 min prior to the beginning of the experiment. Oxygen concentration was measured at time zero and after 30 min, using a portable oxymeter. Oxygen consumption was expressed in milligrams of oxygen per gram of body weight per hour. The eyestalks were ablated as described above.

### 4.6. Experimental Design and Measurements of Melatonin Eyestalk Ganglia in Vitro and in Vivo

In *in vitro* experiments, 10 eyestalks (*n* = 5) were incubated for 45 min at 0.7, 2, 4 or 6 mgO_2_·L^−1^. The eyestalks were then frozen at −80 °C for further analyses.

The frozen optic lobes were homogenized in 0.5 mL ethanol, centrifuged for 10 min (1000× *g*), and the supernatants were collected and lyophilized. After the samples were diluted with phosphate-buffered saline (PBS) and Radioimmunoassay (RIA) buffer (400 µL/sample), they were sonicated for 4–5 s in an ice bath and centrifuged for 5 min (10,000× *g*). The supernatants were directly analyzed for melatonin (2 × 100 µL) as described previously, using radio-iodinated melatonin as the tracer [46,47].

In *in vivo* experiments, 12 aquaria (3 L each) were purged with nitrogen gas to reduce the level of dissolved oxygen to 6, 2 or 0.7 mgO_2_·L^−1^ (4 aquaria each). Forty-eight (*n* = 4) crabs were injected with 100 μL of PS (control) or MEL (20 × 10^−12^, 20 × 10^−9^ or 20 × 10^−7^ M) and exposed to the above oxygen concentrations in the respirometer chamber with saline water (salinity at 20) for 15 min prior to the beginning of the experiment. Oxygen concentration was measured at time zero and after 30 min, using a portable oxymeter. The eyestalks were then frozen at −80 °C for further melatonin quantification as described above.

### 4.7. Statistical Analysis

In the CHH gene expression, the REST^©^ software (Munich, Germany) was applied in the relative quantification [48], with pairwise comparisons. For VO_2_, glucose and lactate, a two-way ANOVA (Analysis of Variance) was carried out followed by an *a posteriori* comparison (Newman–Keuls test, α = 0.05). ANOVA assumptions (normality and homogeneity of variance) were verified prior to ANOVA analysis.

## References

[1] Lerner A.B., Case J.D., Takahashi Y., Lee T.H., Mori W. (1958). Isolation of melatonin, the pineal gland factor that lightens melanocytes. J. Am. Chem. Soc..

[2] Lerner A.B., Case J.D., Heinzelman R.V. (1959). Structure of melatonin. J. Am. Chem. Soc..

[3] Underwood H. (1981). Circadian organization in the lizard *Sceloporus. occidentalis*: The effects of pinealectomy, blinding and melatonin. J. Comp. Physiol..

[4] Mayer I., Bornestaf C., Borg B. (1997). Melatonin in non-mammalian vertebrates: Physiological role in reproduction?. Comp. Biochem. Physiol. A.

[5] Lutterschimidt D.I., Lutterschimidt W.I., Hutchison V.H. (2003). Melatonin and thermoregulation in ectothermic vertebrates: A review. Can. J. Zool..

[6] Reiter R.J. (1991). Melatonin: The chemical expression of darkness. Mol. Cell. Endocrinol..

[7] Vivien-Roels B., Pévet P. (1993). Melatonin: Presence and formation in invertebrates. Experientia.

[8] Maciel F.E., Geihs M.A., Monserrat J.M., Nery L.E.M. (2010). Antioxidant defense system rhythms in crustaceans and possible roles for melatonin. Front. Biosci..

[9] Sainath S.B., Swetha C.H., Reddy P.S. (2013). What do we (need to) know about the melatonin in crustaceans?. J. Exp. Zool. A.

[10] Maciel F.E., Ramos B.P., Geihs M.A., Vargas M.A., Cruz B.P., Meyer-Rochow V.B., Vakkuri O., Allodi S., Monserrat J.M., Nery L.E.M. (2010). Effects of melatonin in connection with the antioxidant defense system in the gills of the estuarine crab *Neohelice granulata*. Gen. Comp. Endocrinol..

[11] Tilden A.R., Mcgann L., Schwartz J., Bowe A., Salazar C. (2001). Effect of melatonin on hemolymph glucose and lactate levels in the ﬁddler crab *Uca pugilator*. J. Exp. Zool..

[12] Sainath S.B., Reddy P.S. (2010). Melatonergic regulation of hemolymph sugar levels in the fresh water edible crab, *Oziotelphusa senex senex*. J. Exp. Zool. A.

[13] Herreid C.F., Full R.J., Burggren W.W., McMahon B.R. (1988). Energetics and locomotion. Biology of Land Crabs.

[14] Spicer J.I., Hill A.D., Taylor A.C., Strang R.H.C. (1990). Effect of aerial exposure on concentrations of selected metabolites in the blood of the Norwegian lobster *Nephrops norvegicus* (Crustacea: Nephropidae). Mar. Biol..

[15] Morris S., Olivier S. (1999). Circulatory, respiratory and metabolic response to emersion and low temperature of *Jasus edwardsii*: Simulation studies of commercial shipping methods. Comp. Biochem. Physiol. A.

[16] Durand F., Devillers N., Lallier F.H., Regnault M. (2000). Nitrogen excretion and change in blood components during emersion of the subtidal spider crab *Maia squinado* (L.). Comp. Biochem. Physiol. A.

[17] Speed S.R., Baldwin J., Wong R.J., Wells R.M.G. (2001). Metabolic characteristic of muscles in the spiny lobster, *Jasus edwardsii*, and responses to emersion during simulated live transport. Comp. Biochem. Physiol. B.

[18] Ridgway I.D., Taylor A.C., Atkinson R.J.A., Stentiford G.D., Chang E.S., Chang S.A., Neil D.M. (2006). Morbidity and mortality in Norway lobsters, *Nephrops norvegicus*: Physiological, immunological and pathological effects of aerial exposure. J. Exp. Mar. Biol. Ecol..

[19] Santos E.A., Nery L.E.M. (1987). Blood glucose regulation in an estuarine crab, *Chasmagnathus granulata* (dana, 1851) exposed to different salinities. Comp. Biochem. Physiol. A.

[20] Spaargaren D.H., Haefner P.A. (1987). The effect of environmental osmotic conditions on blood and tissue glucose levels in brown shrimp, *Crangon crungon* (L.). Comp. Biochem. Physiol. A.

[21] Geihs M.A., Maciel F.E., Vargas M.A., Cruz B.P., Nery L.E.M. (2013). Effects of hypoxia and reoxygenation on the energetic metabolism of the crab *Neohelice granulata* (Decapoda, Varunidae). J. Exp. Mar. Biol. Ecol..

[22] Zou E., Du N., Lai W. (1996). The effects of severe hypoxia on lactate and glucose concentrations in the blood of the Chinese freshwater crab *Eriocheir sinensis* (Crustacea: Decapoda). Comp. Biochem. Physiol. A.

[23] Maciel J.E.S., Souza F., Valle S., Kucharski L.C., Da Silva R.S.M. (2008). Lactate metabolism in the muscle of the crab *Chasmagnathus granulatus* during hypoxia and post-hypoxia recovery. Comp. Biochem. Physiol. A.

[24] Silva-Castiglioni D., Oliveira G.T., Buckup L. (2010). Metabolic responses of *Parastacus defossus* and *Parastacus brasiliensis* (Crustacea, Decapoda, Parastacidae) to hypoxia. Comp. Biochem. Physiol. A.

[25] Maciel F.E. (2010). Papel da melatonina nas respostas metabólicas do caranguejo *Neohelice granulata* (Dana, 1851) (Decapoda; Brachyura). Ph.D. Thesis.

[26] Webster S.G., Keller R., Dircksen H. (2012). The CHH-superfamily of multifunctional peptide hormones controlling crustacean metabolism, osmoregulation, moulting, and reproduction. Gen. Comp. Endocrinol..

[27] Halperin J., Ansaldo M., Pellerano G.N., Luquet C.M. (2000). Bimodal breathing in the estuarine crab *Chasmagnathus granulatus* Dana 1851—Physiological and morphological studies. Comp. Biochem. Physiol. A.

[28] Schmitt A.S.C., Santos E.A. (1993). Behaviour and haemolymphatic ionic composition of the intertidal crab *Chasmagnathus granulata* Dana, 1851 (Crustacea: Decapoda) during emersion. Comp. Biochem. Physiol. A.

[29] D’Incao F., Ruffino M.L., Silva K.G., Braga A.C. (1992). Responses of *Chasmagnathus granulata* Dana (Decapoda: Grapsidae) to salt-marsh environmental variations. J. Exp. Mar. Biol. Ecol..

[30] Santos E.A., Baldisseroto B., Bianchini A., Colares E.P., Nery L.E.M., Manzoni G.C. (1987). Respiratory mechanisms and metabolic adaptations of an intertidal crab, *Chasmagnathus granulata* (Dana, 1851). Comp. Biochem. Physiol. A.

[31] Schmitt A.S.C., Santos E.A. (1993). Lipid and carbohydrate metabolism of the intertidal crab *Chasmagnathus granulata* Dana, 1851 (Crustacea: Decapoda) during emersion. Comp. Biochem. Physiol. A.

[32] Oliveira G.T., Eicheler P., Rossi I.C., Da Silva R.S.M. (2004). Hepatopancreas gluconeogenesis during anoxia and post anoxia recovery in *Chasmagnathus granulata* crabs maintained on high-protein or carbohydrate rich diets. J. Exp. Zool. A.

[33] Geihs M.A., Vargas M.A., Maciel F.E., Caldas S.S., Cruz B.P., Primel E.G., Monserrat J.M., Nery L.E.M. (2010). Effect of melatonin in the antioxidant defense system in the locomotor muscles of the estuarine crab *Neohelice granulata* (Dacapoda, Brachyura). Gen. Comp. Endodocrinol..

[34] Chung J.S., Zmora N. (2008). Functional studies of crustacean hyperglycemic hormones (CHHs) of the blue crab, *Callinectes sapidus*—The expression and release of CHH in eyestalk and pericardial organ in response to environmental stress. FEBS J..

[35] Hoyos M., Guerrero J.M., Perez-Cano R. (2000). Serum cholesterol and lipid peroxidation are decreased by melatonin in diet-induced hypercholesterolemic rats. J. Pineal Res..

[36] Nishida S., Sato R., Murai I., Nakagawa S. (2003). Effect of pinealectomy on plasma levels of insulin and leptin and on hepatic lipids in type 2 diabetic rats. J. Pineal Res..

[37] Peschke E. (2008). Melatonin, endocrine pancreas and diabetes. J. Pineal Res..

[38] Robeva R., Kirilov G., Tomova A., Kumanov P. (2008). Melatonin–insulin interactions in patients with metabolic syndrome. J. Pineal Res..

[39] Zanquetta M.M., Seraphim P.M., Sumida D.H. (2003). Calorie restriction reduces pinealectomy-induced insulin resistance by improving GLUT4 gene expression and its translocation to the plasma membrane. J. Pineal Res..

[40] Richter K., Peschke E., Peschke D. (1999). Effect of melatonin on the release of prothoracicotropic hormone from the brain of *Periplaneta americana* (Blattodea: Blattidae). Eur. J. Entomol..

[41] Huybrechts J., de Loof A., Schoofs L. (2005). Melatonin-induced neuropeptide release from isolated locust corpora cardiaca. Peptides.

[42] Tilden A.R., Brauch R., Ball R., Janze A.M., Ghaffari A.H., Sweeney C.T., Yurek J.C., Cooper R.L. (2003). Modulatory effects of melatonin on behavior, hemolymph metabolites, and neurotransmitter release in crayfish. Brain Res..

[43] Dubocovich M.L. (1995). Melatonin receptors: Are there multiple subtypes?. Trends Pharmacol. Sci..

[44] Boutin J.A., Audinot V., Ferry G., Delagrange P. (2005). Molecular tools to study melatonin pathways and actions. Trends Pharmacol. Sci..

[45] Mendoza-Vargas L., Solís-Chagoyán H., Benítez-King G., Fuentes-Pardo B. (2009). MT2-like melatonin receptor modulates amplitude receptor potential in visual cells of crayﬁsh during a 24-hour cycle. Comp. Biochem. Physiol. A.

[46] Vakkuri O., Lamsa E., Rahkamaa E., Ruotsalainen H., Leppaluoto J. (1984). Iodinated melatonin: Preparation and characterization of the molecular structure by mass and ^1^H NMR spectroscopy. Anal. Biochem..

[47] Vakkuri O., Leppaluoto J., Vuolteenaho O. (1984). Development and validation of a melatonin radioimmunoassay using radioiodinated melatonin as tracer. Acta Endocrinol..

[48] Pfaffl M.W., Horgan G.W., Dempfle L. (2002). Relative expression software tool (REST^©^) for group-wise comparison and statistical analysis of relative expression results in real-time PCR. Nucleic Acids Res..

